# m^6^A methylation modification and immune cell infiltration: implications for targeting the catalytic subunit m^6^A-METTL complex in gastrointestinal cancer immunotherapy

**DOI:** 10.3389/fimmu.2023.1326031

**Published:** 2023-12-15

**Authors:** Chen Peng, Fen Xiong, Xi Pu, Zhangmin Hu, Yufei Yang, Xuehan Qiao, Yuchun Jiang, Miao Han, Deqiang Wang, Xiaoqin Li

**Affiliations:** ^1^ Department of Medical Oncology, Affiliated Hospital of Jiangsu University, Zhenjiang, China; ^2^ Department of Gastroenterology, Affiliated Hospital of Jiangsu University, Zhenjiang, Jiangsu, China; ^3^ Institute of Digestive Diseases, Jiangsu University, Zhenjiang, China

**Keywords:** N 6 -methyladenosine (m 6 A) methylation modification, catalytic subunit m 6 A-METTL complex, METTL3/14, immune cell infiltration, immunotherapy, gastrointestinal cancers

## Abstract

N^6^-methyladenosine (m^6^A) methylation modification is a ubiquitous RNA modification involved in the regulation of various cellular processes, including regulation of RNA stability, metabolism, splicing and translation. Gastrointestinal (GI) cancers are some of the world’s most common and fatal cancers. Emerging evidence has shown that m^6^A modification is dynamically regulated by a complex network of enzymes and that the catalytic subunit m^6^A-METTL complex (MAC)-METTL3/14, a core component of m^6^A methyltransferases, participates in the development and progression of GI cancers. Furthermore, it has been shown that METTL3/14 modulates immune cell infiltration in an m^6^A-dependent manner in TIME (Tumor immune microenvironment), thereby altering the response of cancer cells to ICIs (Immune checkpoint inhibitors). Immunotherapy has emerged as a promising approach for treating GI cancers. Moreover, targeting the expression of METTL3/14 and its downstream genes may improve patient response to immunotherapy. Therefore, understanding the role of MAC in the pathogenesis of GI cancers and its impact on immune cell infiltration may provide new insights into the development of effective therapeutic strategies for GI cancers.

## 1 Introduction

Gastrointestinal (GI) cancers currently rank as the most common and deadliest cancers, accounting for 26% of global cancer incidence and 35% of all cancer-related deaths. The spectrum of GI cancers encompasses esophageal, gastric, liver, pancreatic, and colorectal cancers, manifesting as a complex multi-step process (1). While current GI cancer treatment mainly includes surgery, radiation therapy, chemotherapy, and targeted therapy, the effectiveness has plateaued. In recent years, immunotherapy has emerged as a prominent topic in GI cancer treatment. Immunotherapy refers to the treatment of tumors through the regulation, activation, or suppression of the patient’s immune system, thereby enhancing their anti-cancer immunity (2). Immune Checkpoint Inhibitors (ICIs) targeting the Cytotoxic T Lymphocyte-associated antigen 4 (CTLA-4) or Programmed cell death protein 1 (PD-1) pathways have demonstrated early success in treating various cancers. However, the subset of patients benefiting from ICIs in GI cancers remains relatively limited (3). Currently, researchers are actively exploring immune mechanisms beyond immune checkpoints to improve the response rate and duration of ICI therapy in GI cancer patients. Considerable attention is also directed towards further investigating m^6^A methylation modification to enhance the efficacy of immunotherapy and screen personalized treatment strategies.

N6-methyladenosine (m^6^A) refers to a methylation modification placed on the sixth nitrogen atom of RNA adenine. It is one of the most common RNA modifications in eukaryotic cells (4) and is widely distributed in eukaryotic and viral mRNAs (5). Sequencing analysis of m^6^A has revealed that it is primarily deposited in the protein-coding sequence (CDS), long internal exons, upstream of the stop codon, and in the untranslated region at the 3’ end of mRNA (6–8). m^6^A methylation modifications exhibit dynamic, reversible, and diversified characteristics, regulated by three m^6^A regulators: methyltransferases, demethylases, and binding proteins (9). As a key regulator of transcriptional expression, m^6^A is closely linked to various aspects of cancer, including development, proliferation, growth, invasion, metastasis, immune system evasion, and other oncogenic or inhibitory functions, through its regulation of RNA processing, splicing, nucleation, translation, and other aspects (10). This paper explores the role of Methyltransferase-Like 3/14 (METTL3/14), components of the catalytic subunit m^6^A-METTL complex (MAC), in regulating tumor immune infiltration. It specifically focuses on their potential and current research progress in clinical applications as biomarkers and immunotherapeutic targets for GI cancers.

## 2 m^6^A methyltransferase complex

**Figure 1:**
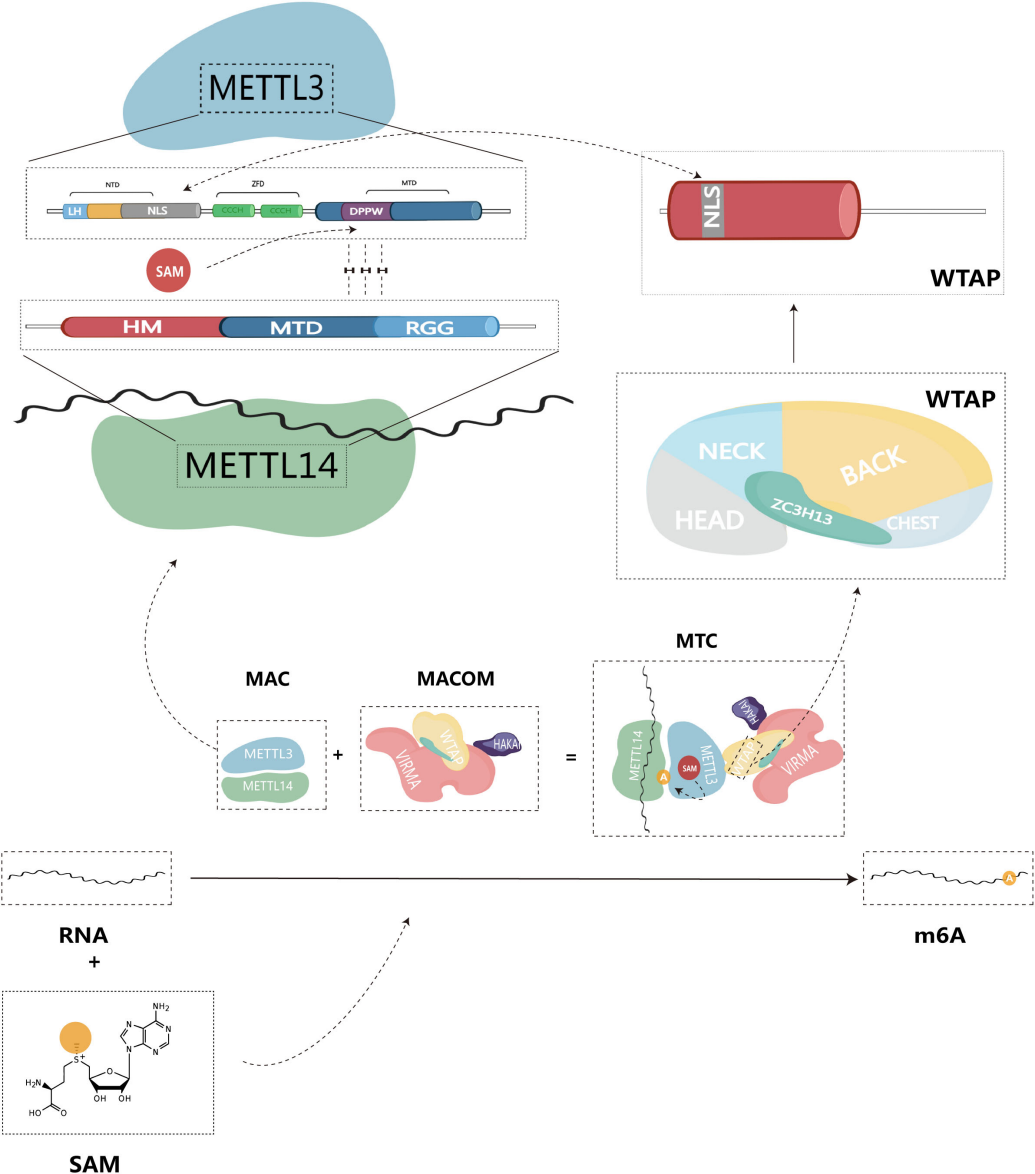
Structure of m6A methyltransferase complex (MTC), and composition of structural domain of MAC-METTL3/14.

The methylation of m^6^A is catalyzed by the m^6^A methyltransferase complex (MTC), also commonly referred to as writers, and it is composed of m^6^A methyltransferases located in the nuclear speckles (11, 12). MTC catalyzes the transfer of methyl groups from the cofactor S-adenosyl-L-methionine (SAM) to the N^6^ end of adenine by binding to SAM (11). The crystal structure of MTC has been determined, revealing its compact globular protein with a highly organized and asymmetric configuration. The active site is located in the hydrophobic pocket at the center of the protein (13). MTC primarily comprises the catalytic subunit, m^6^A-METTL complex (MAC), which is catalytically active, and the regulatory subunit, m^6^A-METTL-associated complex (MACOM), which plays a role in regulating enzymatic activity. This complex is cross-linked through the interaction between Wilms tumor 1-associated protein (WTAP) and METTL3 (**Figure 1**). Subdividing further, METTL3 and METTL14 are the major components of MAC, while HAKAI (also known as CBLL1), WTAP, VIRMA (vir like m6A methyltransferase associated), and ZC3H13 (zinc finger CCCH type containing 13) combine to form MACOM (14).

### 2.1 Catalytic subunit m^6^A-METTL complex

As the first identified m^6^A methyltransferase, METTL3 is a 70 kDa protein with a full-length amino acid count of 580, and it plays a crucial role in catalytic methylation (13, 15). The METTL3 protein consists of three primary structural domains: the N-terminal structural domain (NTD), the zinc finger structural domain (ZFD), and the methyltransferase structural domain (MTD), serving as the primary catalytic structure.

NTD: This structural domain is located at the beginning of the protein sequence and contains a leader helix (LH) and a nuclear localization signal (NLS) region (16). The NTD plays a key role in regulating catalytic activity and substrate specificity. Specifically, the NTD has been shown to influence enzymatic activity by methylating different RNA structures based on their stability and folding patterns, thus altering the dependency of secondary structure on yield levels (17). Additionally, the NTD collaborates with other elements in MAC, such as the RGG structural domain of METTL14, to improve the efficiency of the methyltransferase reaction (17, 18). This synergistic interaction of the NTD with other structural domains in METTL3 highlights the complex interaction of various elements within the enzyme, ultimately governing its catalytic activity and substrate specificity.

ZFD: Located near the C-terminal end of the protein, the ZFD contains two CCCH-type zinc fingers, ZnF1 and ZnF2, connected by an anti-parallel β-sheet (19). This structural domain is responsible for recognizing and binding RNA molecules, particularly the GGACU motif found in the region where methylation occurs. The ZFD stabilizes the protein structure and allows it to interact with RNA molecules, a requirement for maintaining MTC’s enzymatic activity (20).

MTD: The MTD construct of METTL3 spans residues 358-580 and contains the conserved DPPW motif, which is essential for enzyme activity (1). MTD primarily adopts a classical α -β -α sandwich fold structure, comprising a mixed eight-stranded β-sheet, four α-helices, and three 310 helices on either side (13). This region binds to the methyltransferase structural domain of METTL14, forming a heterodimeric complex responsible for the specific recognition of the m^6^A modification site in RNA (19).

In summary, the three structural domains of METTL3 work in concert to ensure the proper recognition, binding, and methylation of RNA molecules, which are essential for the regulation of gene expression and various other cellular processes.

Similar to METTL3, METTL14 also belongs to the family of proteins homologous to the MT-A70 subunit (21). METTL14 is a monomeric protein with a molecular weight of approximately 52 kDa (16). It consists of three structural domains: N-terminal extension (denoted HM), methyltransferase structural domain (MTD), and Arginine-Glycine repeats (RGG) at the C-terminus (22). The RGG repeats of METTL14 can contribute to maintaining the catalytic activity of MTC during methylation. Since the RGG repeats are present in RNA-binding proteins, they can bind to the target RNA, thus playing an important role in the methylation reaction (23). MAC can be directly recruited to specific methylation sites through the preferential and specific binding of RNA G-quadruplex (rG4) structures by the RGG repeats of METTL14 (18). *In vitro*, MAC demonstrates high activity with the RRACH site, preferentially binding to it and methylating it (24). In contrast to METTL3, METTL14 lacks residues that form hydrogen bonds with SAM. Therefore, the MTD domain of METTL14 does not have an active site accommodating donor and acceptor substrates. Instead, it enhances methyltransferase activity by recognizing RNA substrates and methyl localization (13, 24, 25).

METTL3 possesses binding sites for SAM and S-adenosylhomocysteine (SAH), mainly located at the carboxyl terminus of the β1, β7, and β8 chains (19). SAM donor binding occurs through multiple hydrogen bonding interactions. Consequently, METTL3 acts as the primary catalytically active subunit in MAC, while METTL14 mainly contributes to m^6^A modification as a metastable activator of METTL3 activity (26). Given that both METTL3 and METTL14 possess a methyltransferase structural domain (MTD), METTL14 forms a stable heterodimer with the MTD of METTL3 in a 1:1 ratio. METTL14 serves as an RNA binding platform, facilitating MAC’s binding to RNA substrates (13, 19, 24). Therefore, we believe that MAC is a core component mediating m^6^A methylation.

### 2.2 Regulatory subunit m^6^A-METTL-associated complex

As a subunit responsible for regulating enzyme activity, MACOM possesses a stronger RNA binding ability than MAC and can directly bind to RNA substrates (14). In the absence of MACOM, the catalytic activity of MAC is significantly reduced (27). Among its components, WTAP lacks any recognizable structural domain by itself (23). Upon binding to MAC, WTAP can recruit other m^6^A methyltransferases to target RNAs, thus regulating intracellular m^6^A deposition (28). Additionally, WTAP plays a crucial role in localizing MAC to nuclear speckles through the potential nuclear localization signal (NLS) found in both WTAP and METTL3 N terminus (29). VIRMA, also known as KIAA1429, is the largest component of the MACOM complex and forms the core framework of MACOM by cross-linking with WTAP (14). Similar to WTAP, VIRMA is also localized in nuclear speckles (30). VIRMA predominantly acts on the 3’UTR and near-termination codon attachment, selectively and preferentially mediating m^6^A methylation modifications in this region (31). During the methylation process, VIRMA mediates regioselective methylation by recruiting core methyltransferases, METTL3/14. ZC3H13, a key component of MACOM, can induce conformational changes in MACOM through interactions with VIRMA (14). ZC3H13 contributes to the nuclear localization of MACOM by localizing to nuclear patches *via* a low-complexity (LC) region (occupying 80% of the ZC3H13 protein sequence) (32). In the study by Philip et al, ZC3H13 maintains the connection between the RNA-binding protein RBM15 and WTAP. By bridging these factors, ZC3H13 plays a role in regulating the specificity and selectivity of m^6^A modification, ensuring it occurs at the appropriate location in the mRNA (33). HAKAI is not a core of the MACOM structure. It has a flexible but relatively conservative presence in the MACOM complex, serving to maintain the stability and integrity of the MACOM components (14). The ubiquitination domain of HAKAI plays a decisive role in this process (34). By regulating RNA-binding components that interact with the m^6^A methyltransferase complex, HAKAI influences the site-specific placement of m^6^A modifications on mRNA and regulates mRNA stability, translation, and other processes (35).

## 3 MAC and GI cancer progression

Numerous studies have previously demonstrated the close association between MAC and cancer, particularly in GI cancers, where METTL3/14, a component of MAC, exerts various effects on tumor progression, including proliferation, growth, invasion, and metastasis. METTL3/14 can act as either a tumor promoter or a tumor suppressor in GI cancers. Different studies have indicated that METTL3/14 may play opposing roles within the same type of tumor. This variability may be attributed to differences in the targeted genes or pathways (**Table 1**). Given this intricate and paradoxical relationship, a comprehensive understanding of the role of METTL3/14 in tumor immunity is necessary, with a specific emphasis on deciphering the potential mechanisms of METTL3/14 within the TIME.

**Table 1:** Regulation of GI cancer progression by MAC.

Cancer	MAC	Promoter/suppressor	Mechanism
**EC (Esophageal cancer)**	METTL3	promoter	METTL3-promoter P300—HDGF mRNA m^6^A —IGF2BP3—Promoting cancer and liver metastasis through tumor angiogenesis and glycolysis (36)
	METTL14	promoter	METTL14—HDAC2 phosphorylation—CSC persistence and resistance to radiotherapy (37)
**CRC (Colorectal Cancer)**	METTL3	promoter	METTL3—GLUT1 mRNA m^6^A—mTORC1—Promoting cancer (38)
		suppressor	METTL3—p38/ERK—Inhibition of CRC cell proliferation and migration (39)
	METTL14	suppressor	METTL14—YTHDF2—lnc XIST mRNA m^6^A—Inhibition of CRC growth and metastasis (40)
**GC (Gastric cancer)**	METTL3	promoter	METTL3—YTHDF-APC mRNA m^6^A—APC Expression—CCND1/MYC—Promoting cancer (41)
	METTL14	promoter	METTL14—LINC01320—sponged miR-495-5p—RAB19—Promoting proliferation, migration and invasion of gastric cells (42)
		suppressor	METTL14——AKT1S1/EIF4B—Inhibition of proliferation and invasion (43)
**LC (Liver cancer)**	METTL3	promoter	METTL3—IGF2BP2—SOX2 mRNA m^6^A—promoting CRC tumorigenesis and metastasis (44)
	METTL14	promoter	METTL14—circFUT8 m^6^A—CHMP4B—promoting HCC cell malignancy (45)
		suppressor	METTL14—USP48 mRNA—SIRT^6^ ubiquitination—glycolysis—cancer inhibition (46)
**PC (Pancreatic Cancer)**	METTL3	promoter	METTL3—miR-380-3p—PTEN/Akt pathway—accelerating cancer aggressiveness (47)
	METTL14	promoter	METTL14—YTHDF2—PPERP mRNA—promoting the growth and metastasis (48)

## 4 MAC and tumor immune cell infiltration

In recent years, as our understanding of the immune system has expanded, an increasing number of researchers have recognized the important role played by the TIME in various tumor biological processes, including tumor development and metastasis (49). TIME refers to all the immune components within it, which, through their interactions, exert influence on the dynamics and complexity of the tumor microenvironment (50). TIME comprises a diverse array of components, including immune infiltrating cells, cytokines, and regulatory proteins (51). Among these constituents, immune infiltrating cells are the primary components of TIME and include CD8^+^ T cells, CD4^+^ helper T cells, regulatory T cells (Treg), B cells, natural killer cells, myeloid-derived suppressor cells (MDSCs), tumor-associated macrophages (TAMs), tumor-associated neutrophils (TANs), and dendritic cells (**Table 2**). One of the main mechanisms of immunotherapy involves immune checkpoint blockade, which targets inhibitory signals on T cells to enhance their activity against cancer cells (61). Tumor cells can upregulate immune checkpoint molecules, such as PD-L1, which inhibit T cell activity and evade immune surveillance. By blocking these inhibitory signals, ICIs enhance the infiltration and activation of immune cells within the tumor microenvironment (62). The level of immune cell infiltration within the tumor microenvironment also plays a key in determining the response to immunotherapy. Tumors with high levels of T-cell infiltration, often referred to as “hot” tumors, are more likely to respond to immunotherapy compared to tumors with low levels of infiltration known as “cold” tumors (63). Therefore, strategies aimed at increasing immune cell infiltration in tumors, including combination therapies that target the tumor microenvironment, are under investigation to improve the response to immunotherapy. In this context, we provide a detailed description of the regulation of immune infiltrating cells within tumors by MAC.

**Table 2:** Function of immune cells in TIME.

Name	Function	Description
**CD8^+^ T**	Effector cells	CD8^+^ T cells express MHC class I on their surface and can specifically recognize tumor-associated antigens. Upon binding to tumor cells, CD8^+^ T cells release perforins and other cytotoxins that can induce apoptosis of tumor cells (52).
**CD4^+^Th**	Synergistic cells	CD4^+^ Th can differentiate into different cell subtypes, which include Th1, Th2, Th17, Th9, Treg, Tfh etc. By secreting multiple cytokines CD4^+^ T cells can activate CTL and enhance its virulence-killing activity (53).
**Treg**	Immunosuppressive cells	In the tumor microenvironment, Treg is mainly involved in suppressing inflammatory responses and producing immunosuppression and is an important subset of T cells involved in immune regulation and self-tolerance (54).
**B cell**	Humoral immune cells	Upon activation by antigen B cells produce plasma cells with antigen-specific antibodies, thereby mediating humoral anti-tumor immunity (55).
**NK cell**	Cytotoxic effector cells	NK cells are able to recognize and eliminate tumor cells by releasing perforins, granzymes and cytokines, and are also efficient producers of interferon-γ (IFN-y) (56).
**MDSC**	Immune escape cells	MDSCs are heterogeneous populations of immature myeloid cells that secrete a variety of immunosuppressive factors and play a critical role in suppressing T cell-mediated immune responses (57).
**TAM**	Scavenger cells	TAMs can phagocytose and remove cellular debris, maintain tissue homeostasis and defend against infection. In the tumor microenvironment, macrophages often help tumor growth and metastasis and escape from immune cells.
		M1 type → suppress tumor, promote inflammation and immune activity;
		M2 type → promotes tumor, tissue repair and immune escape (58).
**TAN**	Inflammatory response cells	TANs play a critical role in carcinogenesis, cell proliferation, cancer-associated inflammation, immunosuppression and epithelial-mesenchymal transition (EMT). TANs release various inflammatory cytokines and chemokines to promote tumor growth and metastasis (59).
**DC**	Antigen-presenting cell	DCs are specialized antigen-presenting cells (APCs) that play a critical role in processing and presenting antigens to activate the T-cell immune response (60).

### 4.1 T cells

METTL3 plays a crucial role in maintaining the proper balance of T cells *in vivo*. Mechanistically, METTL3-dependent m^6^A methylation of specific mRNA transcripts within T cells can modulate the stability and translation of these transcripts, leading to changes in gene expression that are critical for T cell survival, proliferation, and differentiation. In particular, METTL3/14 promotes the degradation of SOCS mRNA and reduces the expression of SOCS family proteins through mediated methylation of SOCS family mRNAs such as SOCS1, SOCS3, and CISH. This leads to sustained activation of the IL-7/STAT5 signaling pathway, which promotes T-cell differentiation and proliferation (64). A recent study identified the effect of METTL3 on the expression of PD-L1. They found that METTL3 mediated the m^6^A modification of PD-L1 mRNA, leading to increased expression of PD-L1. When PD-L1 binds to PD-1 on T cells, this elevated expression of PD-L1 suppresses the immune response against tumors by curbing T cell activation and function (65). This process can be regulated by JNK signaling (66). Wang and colleagues share a similar perspective, suggesting that METTL3/14-mediated m^6^A modifications affect the stability and translation efficiency of Stat1 and Irf1 mRNAs. This, in turn, results in the suppression of CD8^+^ T cell infiltration within the TIME, resulting in decreased secretion of cytokines such as IFN-γ (67). Therefore, based on the aforementioned study, Wang et al. discovered that the phytochemical Delicaflavone inhibits the expression of METTL3/14, subsequently reducing the secretion of cytokines and chemokines. This inhibition also led to the downregulation of STAT1-IRF1, thereby activating antitumor immunity. Additionally, it triggers oxidative stress, amplifies the recruitment of CD8^+^ T cells into tumor tissues, increases the production of Th1 cytokines, and ultimately inhibits tumor growth (68).

Treg, as a subset of T cells, plays an essential role within the TIME by suppressing the immune response of other T cells and secreting immunosuppressive factors that promote tumor escape (54). Conversely, the deletion of METTL14 expression in T cells disrupts the differentiation of naive T cells into Treg cells, resulting in increased secretion of Th1 and Th17 cytokines (69). Tong et al. elevated SOCS mRNA and protein levels through the inhibition of METTL3-mediated m^6^A modification in regulatory T cells. This resulted in the inhibition of the JAK-STAT signaling pathway, causing Treg to lose its ability to suppress effector T cells and naive T cells (70). Similar findings were observed in METTL14, where the knockdown of METTL14 inhibited Treg cell growth while promoting the infiltration of CD4^+^ and CD8^+^ cells (71). Additionally, METTL3-mediated modification of circQSOX1 leads to the sponging of miR-326 and miR-330-5p, thus promoting PGAM1 expression. This process leads to an upregulation of glycolysis levels and increased lactate production in colorectal cancer (CRC) cells. The accumulation of lactate within the TIME facilitates Treg infiltration while suppressing the infiltration of CD8^+^ T cells, ultimately promoting immune evasion (72).

T follicle helper (Tfh) cells, a subset of CD4^+^ T cells, are responsible for directing and activating tumor-specific B cells and T cells, thereby promoting the formation and enhancement of anti-tumor immunity. However, tumors can exploit these cells to suppress the immune response, promoting tumor growth and metastasis (73). METTL14 promotes Th-17 cell differentiation by mediating m^6^A methylation of exosomal miR-149-3p (74). Similarly, METTL3 promotes TFH differentiation by stabilizing transcripts of key TFH signature genes, such as Tcf7, and mediating their m^6^A methylation (75). A study by Zhu et al. elucidated the mechanism by which the E3 ubiquitin ligase VHL regulates Tfh cell differentiation. Specifically, the authors identified GAPDH as a key player in Tfh cell differentiation and demonstrated that VHL deficiency leads to the upregulation of GAPDH. This, in turn, led to reduced expression of the Tfh cell marker ICOS and attenuated Tfh cell differentiation, attributed to METTL3/14-mediated modification of ICOS mRNA m^6^A. This mechanism plays a crucial role in regulating metabolic reprogramming during the early stages of Tfh cell differentiation, with the HIF-1α axis serving as the primary regulator (76). Th1 and Th2 cells are the two distinct classes of T cells involved in immune responses. Th1 cells are responsible for rejecting foreign molecules, whereas Th2 cells contribute to tolerance toward foreign molecules. Knockdown of METTL3 effectively counteracts LPS-induced Th1 differentiation while significantly increasing the proportion of Th2 (77).

### 4.2 B cells

Currently, there exists limited research exploring the mechanisms through which METTL3 regulates B cells within the tumor microenvironment. Some studies have suggested that METTL3 has only marginal effects on B cell development in normal tissue environments (78). However, Zheng’s study demonstrated that METTL3/14 is necessary for normal B-cell development because it induces cell proliferation of Pro-B and facilitates the differentiation of Pro-B into large-Pre-B/small-Pre-B by promoting IL-17 secretion and IL-17 receptor expression. Additionally, the activation of key transcription factors such as IKZF3, which promotes the conversion of large Pre-B to small Pre-B, is intricately linked to METTL14 (79). Moreover, METTL14-mediated m^6^A modification upregulates genes crucial for the germinal centre response, thus positively contributing to the positive selection and proliferation of germinal centre B cells (80). While these investigations weren’t specifically carried out in a tumor setting, they offer valuable insights into how METTL3/14 regulates B cells. These findings hint at how METTL3/14 could potentially influence B cells within the tumor microenvironment, conducting additional experiments is imperative to gain a comprehensive understanding of the precise mechanisms by which METTL3/14 governs B cell behavior within this specific context.

### 4.3 DCs

Dendritic cells play an important immunoregulatory role within the tumor microenvironment. They capture and process antigens from tumor cells and secrete various cytokines, thereby activating T cell-mediated anti-tumor immune responses (60). METTL3 knockout impairs DC activation, characterized by reduced surface expression of costimulatory molecules, decreased cytokine production, and impaired T-cell activation. Further mechanistic studies suggest that METTL3 mediates m6A methylation modification of key signaling molecules (Tirap, CD40, and CD80) involved in DC activation and function. This enhances the stability and translational efficiency of these signaling molecule mRNAs, ultimately promoting DC activation and maturation, and improving DC function both *in vitro* and *in vivo* (81). Wu et al. also observed significantly higher levels of METTL3 mRNA and protein in mature DCs compared to immature DCs. Knocking down the METTL3 gene in DCs resulted in reduced expression of related cytokines, such as IFN-γ and IL-12, and reduced T cell proliferation, ultimately inducing immune tolerance (81).

### 4.4 NK cells

The expression of METTL3 plays a pivotal role in the anti-tumor immunity of NK cells, which are core immune cells responsible for cytotoxic effects. METTL3 promotes elevated expression of SHP-2, a key ERK activator, by mediating the m^6^A modification of PTPN11. This, in turn, promotes IL-15 signaling, and in turn, leads to increased proliferation and enhanced effector functions of NK cells (82).

### 4.5 Macrophages

When tumor cells evade clearance and surveillance by macrophages, these macrophages polarize and differentiate into two subtypes: M1 and M2. M1 macrophages are capable of killing tumor cells and recognizing foreign antigens, while M2 macrophages produce immunosuppressive factors in the tumor microenvironment, acting as suppressors of the immune response (58). Defective expression of METTL14 on macrophages results in increased EBI3 expression, leading to CD8^+^ T cell dysfunction (83). Liu et al. suggested that METTL3 modifies STAT1 mRNA through m^6^A methylation, influencing the expression of genes associated with the M1 macrophage phenotype and decreasing the expression of genes associated with the M2 macrophage phenotype. This makes METTL3 a key regulator of macrophage polarization and function (84). Tong et al. also suggested that METTL3 positively regulates M1 macrophages primarily by targeting IRAKM mRNA, promoting its degradation and thereby regulating TNF-α levels through TLR4 signaling in macrophages (85). Furthermore, inhibiting METTL3 activity with LXA4 promotes macrophage polarization toward the M2 phenotype (86). Shu et al. proposed an innovative signaling axis consisting of METTL3/MALAT1/PTBP1/USP8/TAK1, where METTL3-mediated m^6^A modification of MALAT1 leads to macrophage pyroptosis by interacting with PTBP1, subsequently triggering downstream signals, including USP8 and TAK1 (87). Recent studies by Yin et al. have shown that METTL3 depletion in macrophages activates the NF-κB pathway and STAT3 signaling, resulting in increased infiltration of M1 and M2 TAMs and Treg cells into the tumor, ultimately remodeling the tumor microenvironment (88). In response, M2-TAMs significantly enhance METTL3 expression in lung adenocarcinoma cells, leading to increased m^6^A methylation levels and overall levels of immune resistance, thus promoting tumor progression (89).

### 4.6 MDSCs

MDSCs are a type of immune cell that exhibits strong immunosuppressive effects. They employ various pathways to inhibit the body’s immune response within the TIME (57). Studies have revealed a positive correlation between the density of CD33^+^ MDSCs in tumor tissues and METTL3 expression (90). Research indicates that METTL3 plays a crucial role in advancing MDSC migration in colorectal cancer by exerting control over the m^6^A-BHLHE41-CXCL1/CXCR2 axis. This involves METTL3 reducing the expression of BHLHE41, a transcription factor associated with tumor immunity suppression, by methylating m^6^A sites on its mRNA. Consequently, this promotes the migration of MDSCs to tumor sites (91). In clinical practice, arterial infusion chemotherapy with cisplatin has been shown to reduce the methylation status of METTL3-regulated genes and decrease MDSCs in tumors (92). This suggests the potential for combining immunotherapy and chemotherapy in cancer treatment.

Derived from the bone marrow, tumor-infiltrating myeloid cells (TIMs) can indirectly influence MDSC differentiation and function by regulating the activity of immune cells within the TIME. METTL3 expression was found to be upregulated in various tumor tissues, particularly in TIMs compared to normal adjacent tissues. The loss of the METTL3 gene in myeloid cells led to a reduction in the infiltration of exhausted CD8^+^ T cells, a decrease in the number of Treg cells, and an increase in the number of tumor-infiltrating CD8^+^ T cells (93).

### 4.7 TANs

Tumor-associated neutrophils (TANs) can adopt various roles within the tumor microenvironment, including promoting tumor growth and metastasis and suppressing immune cell activity (59). Elevated METTL3 expression progressively promotes the expression of c-Rel, a transcription factor involved in immune response regulation, leading to increased IL-8 production and enhanced infiltration of TANs. This, in turn, results in a more robust immune response against cancer cells (94). When TANs are activated by cancer cells in the tumor microenvironment or by inflammatory factors such as G-CSF/IL-8, they undergo a process known as NETosis, where they release DNA and other granular proteins into the extracellular space, forming Neutrophil extracellular traps (NETs) (95). By releasing NETs, neutrophils participate in the immune defense against tumors, yet this action potentially contributes to immunotherapy resistance and immune suppression. Nevertheless, the removal of NETs or the application of specific treatment strategies like DNase I or adeno-associated virus (AAV) vector could enhance immunotherapy efficacy, thereby preventing tumor recurrence (96). Qu et al. suggested that METTL3-mediated modification of SIRT1 mRNA m^6^A leads to autophagosome formation and impaired autophagic flux, regulating the formation and release of NETs (97). Another study found that METTL3 promotes NET-induced cellular iron death by mediating the m^6^A modification of GPX4 (98).

## 5 MAC and GI cancer immunotherapy

**Figure 2:**
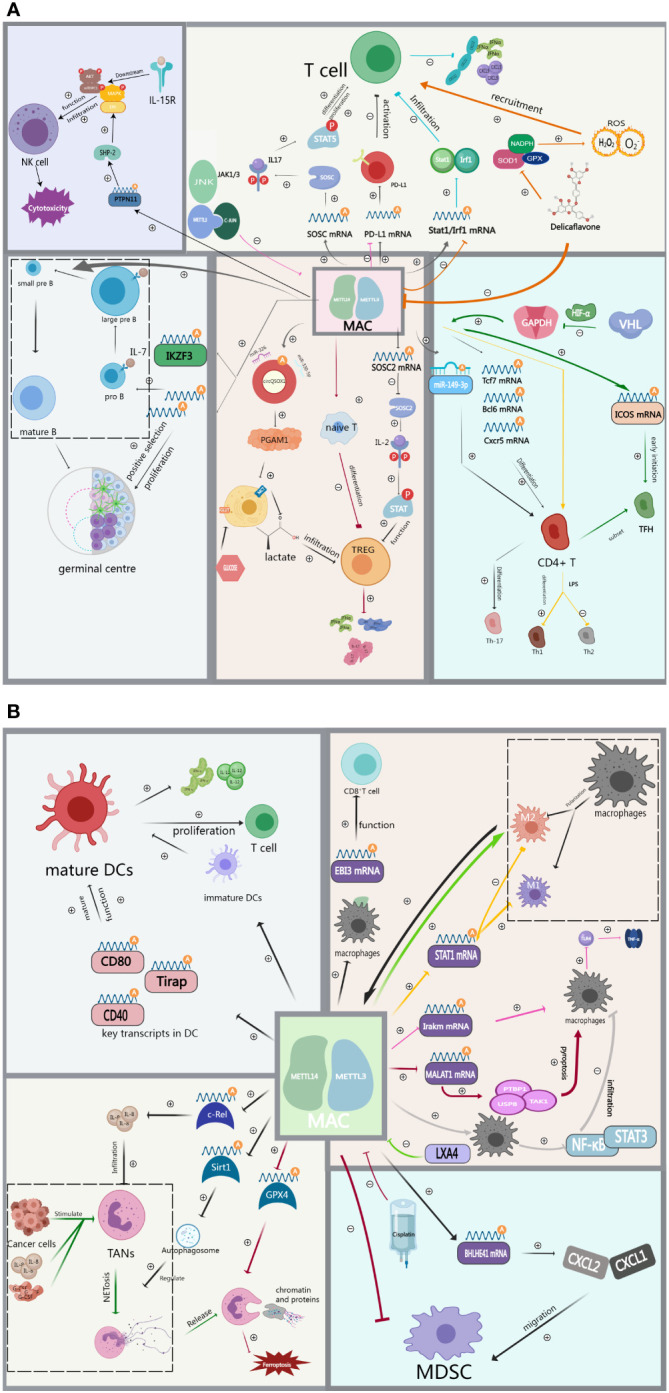
**(A)** MAC regulates immune cell infiltration including T cells, Th cells, TREG, B cells and NK cells in the tumor immune microenvironment. **(B)** MAC regulates immune cell infiltration including DCs, TAMs, TANs and MDSCs in the tumor immune microenvironment.

Although ICIs have significantly improved the prognosis of patients with advanced GI cancers, a significant proportion of patients remain unresponsive to ICIs. Emerging evidence indicates that both intrinsic factors of tumor cells (e.g., immune checkpoints expression levels, tumor mutational load) and external factors (e.g., immune cells infiltration levels within the TIME), are closely associated with ICIs efficacy (99). Therefore, a deeper exploration of the impact of MAC on the TIME (**Figure 2**), including immune cell infiltration levels and immune checkpoint expression such as PD-1, can provide valuable insights for developing novel immunotherapies.

### 5.1 Esophageal cancer

Ranked as the eighth most common cancer type worldwide, the overall prognosis of EC patients under current conventional treatments remains unfavorable. Immunotherapy has shown promise in enhancing the survival of advanced esophageal cancer patients. However, the clinical efficacy of current ICI monotherapy is limited due to the complex immune evasion mechanism involved in EC (100). Guo et al. studied 20 m^6^A methylation regulators and discovered a significant negative correlation between the expression levels of METTL3/METL14 and PD-L1 in patients with esophageal squamous cell carcinoma (ESCC) (101). Additionally, Zhou et al. found that METTL3 expression levels negatively correlated with the infiltration levels of T lymphocytes, B lymphocytes, and neutrophils in ESCC, suggesting that METTL3 plays a key role in the immune regulation of esophageal cancer (102). Therefore, MAC could potentially enhance the sensitivity of ESCC patients to immunotherapy by modulating the TIME and PD-L1 expression. Moreover, shRNA knockdown experiments demonstrated that IFIT2 is a downstream target gene regulated by METTL3 and is closely associated with prognostic immune infiltration. Both factors can serve as immunotherapy targets (103); and prognostic or diagnostic indicators for esophageal cancer, offering novel insights for immunotherapy selection in ESSC patients.

### 5.2 Gastric cancer

Previous clinical studies have demonstrated the clear therapeutic potential of immunotherapy for patients with advanced gastric cancer. However, the highly heterogeneous nature of GC makes the selection of sensitive predictive biological targets a primary focus in current immunotherapy research (104). Zhang et al. classified gastric cancer samples into three categories based on the expression levels of 21 m^6^A regulators through unsupervised clustering analysis. These categories corresponded to three distinct tumor immune patterns: the immune-rejection phenotype, immune-inflammatory phenotype, and immune desert phenotype. These patterns can predict the intensity of the anti-tumor immune response in patients (105). Subsequently, METTL3/14 was found to be highly expressed in m^6^ACluster B, representing an immunoinflammatory phenotype associated with adaptive immune activation. Notably, patients with microsatellite instability (MSI), characteristic of m^6^ACluster B, tend to exhibit better immunotherapy efficacy (106).

METTL3, a major component of MAC, has been shown to influence the response to immunotherapy in GC patients through METTL3-mediated m^6^A methylation. This is evident when analyzing its downstream methylation core molecules (107). Similarly, it was observed that immune cells such as CD8^+^ T cells and T follicular helper cells were significantly more abundant in patient population-Cluster 2, characterized by overall high METTL3/14 expression. Additionally, METTL3 was associated with elevated PD-L1 expression in gastric cancer cells (108). In summary, this study suggests that quantitative analysis of METTL3/14 and its downstream molecules could help in predicting the response of gastric cancer patients to immunotherapy. The potential of using METTL3/14 as a direct target for anti-tumor immunotherapy in gastric cancer patients to promote treatment efficacy warrants further exploration.

### 5.3 Liver cancer

LC continues to have a persistently high mortality rate worldwide due to its unique vascular and neurological distribution. Immunotherapy offers hope for patients with advanced liver cancer who have limited treatment options such as surgery, liver transplantation, and local ablative therapy (109). However, overcoming LC patient resistance to immunotherapy remains a pressing challenge. Gu et al. found a negative correlation between the expression of writers represented by METTL3/14 and the infiltration of pDC, DC, and cytotoxic cells in Hepatocellular carcinoma (HCC). They concluded that METTL3 expression is positively correlated with sensitivity to multiple immune drugs, suggesting potential biomarkers for drug screening (110). Conversely, Jiang et al. discovered a significant correlation between the expression of METTL3/14 and immune checkpoint markers such as PD-L1 and CTLA4. This implies that METTL3/14 could serve as a novel biomarker for predicting the response to anti-PD-1/L1 immunotherapy in HCC (111, 112).

Zhen et al. reported that ZNF320, a member of the Kruppel-like zinc finger gene family, can regulate immune cell infiltration in HCC by affecting the expression levels of related chemokines through positive regulation of METTL3 Correlations with immune checkpoint-related genes revealed that patients with high ZNF320 expression also had elevated PDL-1 expression, suggesting potentially improved anti-PDL-L1 immunotherapy efficacy (113). In contrast, Peng et al. conducted lipopolysaccharide induction experiments in HCC cells and found that high METTL14 expression mediates m^6^A methylation of MIR155HG. MIR155HG can regulate PD-L1 expression through RNA binding protein ELAVL1 or *via* the miR-223/STAT1 axis, potentially affecting immunotherapy (114). Cholangiocarcinoma (CCA), the second most common form of liver cancer, is characterized by its high recurrence rate, low survival rate, and poor prognosis (115). In a recent study by Zhen et al., METTL14 was found to induce the degradation of SIAH2 mRNA and protein in a YTHDF2-dependent manner in cholangiocarcinoma. Subsequent *in vitro* and *in vivo* experiments revealed that SIAH2 inhibits T-cell expansion and cytotoxicity by enhancing the K63-linked ubiquitination of PD-L1 and its interaction with PD-L1, consequently reducing the efficacy of immunotherapy in patients (116). In summary, m^6^A and other related epigenetic modifications can reactivate immunotherapy-related genes in combination with immune therapy. The expression of METTL3/14 can synergize with ICI therapy by regulating METTL3/14 expression, potentially enhancing the anti-tumor response. This holds significant promise for improving the clinical efficacy of immunotherapy in HCC patients.

### 5.4 Pancreatic cancer

Pancreatic cancer (PC), characterized by a poor prognosis, continues to pose a significant clinical challenge. Classical therapies, including chemotherapy, radiotherapy, and targeted therapy have limited long-term benefits for patients. Furthermore, early-stage pancreatic cancer often exhibits suppressed tumor immunity, hindering the effectiveness of immunotherapy in improving survival outcomes for PC patients (117). Liu et al. found that in pancreatic ductal adenocarcinoma (PDAC), high METTL3/14 expression was significantly and positively associated with the enrichment of activated NK cells and mast cells (118). Specifically, within the CD4^+^ T cell subpopulation, METTLE3 was found to be negatively correlated with Th2 and Th17 cells, which regulate inflammatory responses, while it was positively correlated with Th1 and Tfh cells, known for their crucial role in tumor immunotherapy. This suggests that METTL3/14 could potentially regulate the immune microenvironment in pancreatic ductal adenocarcinoma. Wang et al. constructed an m^6^A score model using METTL3 as a prognostic protective gene and found that patients with low scores exhibited higher Immune cell Proportion Scores (IPS) for both anti-PD-1/CTLA-4 therapy alone and immune combination therapy (119). This study underscores the potential of METTL3/14 as a predictor of immunotherapy response in pancreatic cancer patients.

Additionally, Song et al. suggested that LncRNA MALAT1 could serve as a downstream target of METTL3. Inhibiting the expression of METTL3 would lead to a further reduction in MALAT1 expression in pancreatic cancer cells, consequently inhibiting the expression of PD-L1 (120). Conversely, METTL3 increases the expression level of circMYO1C and induces its cyclization. circMYO1C, through its binding to the 3’UTR of PD-L1 mRNA *via* IGF2BP2 protein at its methylation site, stabilizes PD-L1 expression. This ultimately promotes tumor growth by increasing the expression of immunosuppressive molecules such as PD-L1, promoting immune evasion by pancreatic cancer cells (121). Therefore, the direct targeting of m^6^A-modifying regulators to modulate immune checkpoint expression emerges as a potential strategy to improve the efficacy of immunotherapy and extend the prognosis of pancreatic cancer patients.

### 5.5 Colorectal cancer

CRC presents a complex profile and wide range of mutational characteristics. For this reason, immunotherapy has not shown good results in other types of CRC despite yielding satisfactory outcomes in some patients (122). Therefore, there is a need to find effective combinations with enhanced response to immunotherapy in patients with other types of CRC. In Xiong’s study, METTL3 improved immunosuppressive function of myeloid cells by mediating m^6^A modification of JAK1 mRNA and promoting its protein translation (93). Chen et al. reported that METTL3 can regulate the BHLHE41-CXCL1/CXCR2 axis in an m^6^A-dependent manner to promote MDSC migration. Based on this theory, inclusion of METTL3 inhibitors in anti-PD1 therapy showed improved sensitivity of CRC patients to anti-PD1 therapy (93). In addition, as another immune checkpoint inhibitor, anti-CTLA-4 therapy is currently used to treat CRC. The METTL3-mediated m^6^A modification of circQSOX1 results in heightened glycolysis levels and elevated lactate production in CRC cells. This, in turn, facilitates immune evasion by CRC cells. Consequently, the inhibition of these processes, in conjunction with anti-CTLA-4 therapy, holds the potential to diminish resistance to immunotherapy in CRC patients (72).

As another component of MAC, deletion of METTL14 expression in macrophages leads to insufficient activation of CD8^+^ T cell function, which in turn inhibits T-cell anti-tumor responses (83). This concept can be used to guide the development of potential immunotherapies against macrophages. In rectal cancer, Cai et al. found that downregulation of METTL14 resulted in reduced immune cell infiltration indicating that it may be used as an immune infiltration-related prognostic biomarker in rectal cancer (123). On the other hand, it was found that knockdown of METTL3/METTL14 in CRC cells increased CD8^+^ T cell infiltration and promoted IFN-γ, CXCL9 and CXCL10 cytokine secretion, leading to enhanced patient’s response to anti-PD-1 therapy (67). In summary, optimizing the response of CRC patients to ICIs by directly targeting METTL3/14 or its downstream mechanisms may be a new avenue for developing future immunotherapy of colorectal cancer.

## 6 Discussion

Given the role of m^6^A modifications in the regulation of gene expression, the catalytic subunit m^6^A-METTL complex (MAC)-METTL3/14 has emerged as a key player in various biological processes, including cancer development and progression. Recent studies have shown that METTL3/14 can regulate immune cell infiltration in the TIME, highlighting its importance in cancer immunology. In particular, the expression levels of METTL3/14 have been observed to regulate immune function in individuals with GI cancers and impact the effectiveness of tumor immunotherapy. These findings support the clinical potential of METTL3/14 as an immune marker for GI tumors. However, investigations into m^6^A methylation in immunotherapy are still in early stages and many questions remain to be answered. Further studies on METTL3/14 and its downstream mechanisms are needed to provide new possibilities for developing immune precision therapy for GI cancers.

## Author contributions

CP: Writing – original draft, Writing – review & editing. FX: Writing – original draft. XP: Writing – original draft. ZH: Writing – review & editing. YY: Writing – review & editing. XQ: Data curation, Writing – review & editing. YJ: Investigation, Writing – review & editing. MH: Investigation, Writing – review & editing. DW: Writing – review & editing. XL: Writing – review & editing.
